# Concurrent expression of glucose-6-phosphate dehydrogenase and secreted phosphoprotein 1 characterizes an aggressive and immunosuppressive tumor state in hepatocellular carcinoma

**DOI:** 10.3389/fcell.2026.1851314

**Published:** 2026-08-28

**Authors:** Guangjie Han, Lili Mi, Jianfei Shi, Jinfeng Wang, Fei Yin

**Affiliations:** Department of Gastroenterology, The Fourth Hospital of Hebei Medical University, Shijiazhuang, Hebei, China

**Keywords:** hepatocellular carcinoma, pentose phosphate pathway, prognostic biomarker, secreted phosphoprotein 1, single-cell transcriptomics, tumor immune suppression, tumor microenvironment

## Abstract

**Introduction:**

Despite improved outcomes with combined hepatic arterial infusion chemotherapy (HAIC) and systemic therapy in hepatocellular carcinoma (HCC), therapeutic resistance is frequently driven by the tumor microenvironment. We aimed to explore biological features associated with therapeutic resistance in HCC.

**Methods:**

We integrated single-cell transcriptomics of a therapy-progressive lesion with large-scale bulk transcriptomic modeling and independent clinical validation. We performed immunohistochemistry in an independent retrospective cohort of 114 treatment-naïve patients to clinically validate the computational findings.

**Results:**

Single-cell analysis revealed concurrent upregulation of glucose-6-phosphate dehydrogenase (G6PD) and secreted phosphoprotein 1 (SPP1) in tumor hepatocytes from an index therapy-progressive lesion. A metabolic-transcriptional risk signature effectively stratified overall survival in the TCGA-LIHC and ICGC LIRI-JP cohorts. This signature was associated with an “inflamed-yet-suppressed” tumor microenvironment, characterized by concurrent upregulation of multiple immune checkpoints and defective cytotoxic capacity. Exploratory intercellular communication modeling inferred that surviving hepatocytes were major senders of SPP1-related signaling. In the clinical validation cohort, tissue G6PD H-score was significantly associated with shorter recurrence-free survival in univariable analysis (HR = 1.086 per 10-point increase, 95% CI 1.015–1.162, P = 0.017) and exploratory stratified analyses. G6PD was positively correlated with SPP1 at both the bulk transcriptomic and tissue protein levels (protein Spearman r = 0.571, P < 0.0001).

**Discussion:**

These findings suggest that concurrent G6PD and SPP1 expression characterizes an aggressive and immunosuppressive tumor state in HCC. Although this association may provide biological insight into treatment refractoriness, its predictive value for modern combination therapies requires prospective validation in treated cohorts.

## Introduction

Hepatocellular carcinoma (HCC), the predominant form of primary liver cancer, continues to impose a significant mortality burden worldwide, largely attributable to late-stage diagnosis that restricts curative interventions (Bray et al., 2024; Llovet et al., 2022). The therapeutic landscape has been revolutionized by combinations of immune checkpoint inhibitors (ICIs) and anti-angiogenic agents. Multiple phase III trials have validated this approach by demonstrating survival benefits over previous standards (Cheng et al., 2022; Ren et al., 2021; Qin et al., 2023). Furthermore, incorporating hepatic arterial infusion chemotherapy (HAIC)—a locoregional modality delivering high-concentration cytotoxic stress—into systemic backbones has demonstrated profound downstaging potential, alongside emerging mechanistic evidence supporting antitumor immune reconstitution (Xing et al., 2025). Clinically, these combination strategies have yielded high objective response and conversion-to-resection rates, while also conferring meaningful survival benefits in advanced disease settings (Li et al., 2024a; Gao et al., 2025; Zhang et al., 2023). However, despite these advances, a substantial proportion of patients fail to achieve durable responses, and the biological basis of resistance to these intensive regimens remains incompletely understood.

The immune-suppressive tumor microenvironment (TME) drives therapeutic failure in HCC. High-resolution single-cell transcriptomic studies show that advanced HCC microenvironments are highly inflamed yet functionally impaired, characterized by pervasive T cell exhaustion and high expression of immune checkpoint molecules (Zhang Q. et al., 2019). Profiling of therapy-resistant lesions has indicated that this hostile microenvironment continuously reprograms during treatment to suppress cytotoxic effector activity (Li et al., 2024b). Beyond immune cell dysfunction, accumulating evidence suggests that tumor-intrinsic metabolic reprogramming actively contributes to this phenotype. Glucose-6-phosphate dehydrogenase (G6PD) controls flux through the pentose phosphate pathway (PPP), generating NADPH to sustain antioxidant defenses and nucleotide biosynthesis. These functions enable tumor cells to tolerate the genotoxic and oxidative stresses imposed by chemotherapy and targeted agents (TeSlaa et al., 2023; Patra and Hay, 2014). Recent evidence links G6PD upregulation directly to oxaliplatin resistance in HCC via an epigenetic regulatory axis (Jin et al., 2025). Moreover, silencing G6PD in HCC cells promoted CD8^+^ T cell expansion and increased interferon-γ production in co-culture assays (Qu et al., 2025). Collectively, these results implicate PPP-dependent metabolic reprogramming in promoting treatment tolerance and shaping immune responses.

Tumor cells not only adapt intracellular metabolism but also actively remodel the TME through intercellular signaling and epigenetic modification. SPP1 (Secreted Phosphoprotein 1, osteopontin) has emerged as a key intercellular mediator that links tumor hepatocyte programs to broader immunosuppressive remodeling. SPP1-expressing macrophages, which are enriched in immune-excluded, therapy-resistant HCC, directly drove CD8+T cell exhaustion via SPP1-CD44 interactions (Trehan et al., 2025; Jin et al., 2024), and SPP1 signaling coordinated macrophage polarization, fibroblast activation, and drug resistance through fatty-acid metabolic reprogramming (Wang and Wang, 2025). At the same time, lactate-driven lysine lactylation has emerged as a post-translational modification that links Warburg-type metabolic reprogramming to transcriptional regulation and immune evasion in cancer (Zhang D. et al., 2019; Li et al., 2025). These observations provide a conceptual framework linking metabolic states with transcriptional and microenvironmental regulation. However, how metabolic adaptation, lactylation-associated programs, and intercellular signaling are coordinated under therapeutic pressure in HCC remains unclear.

To explore the interplay between metabolic adaptation and microenvironmental remodeling under therapeutic pressure, we adopted a discovery-to-validation approach. First, recognizing the clinical challenges in obtaining post-treatment clinical specimens from advanced HCC, we utilized an exploratory single-cell RNA sequencing (scRNA-seq) analysis of a rare index case with therapy-progressive disease (following HAIC plus anti-PD-1 and TKI therapy) alongside treatment-naïve controls to generate initial biological hypotheses. To prioritize candidates associated with active metabolic-transcriptional states, we employed a curated framework of 462 lactylation-related genes as a bioinformatic filter. This cross-platform integration yielded a five-gene metabolic-transcriptional signature (BZW1, RAN, NR6A1, G6PD, APOC2). Recognizing the statistical limitations of a single-patient discovery cohort, we hypothesized that rather than representing a purely acquired, therapy-induced resistance mechanism, this signature captures a generalized, aggressive tumor state. We subsequently validated its prognostic utility and immunological implications across multiple bulk transcriptomic cohorts (TCGA-LIHC and ICGC LIRI-JP). Finally, we corroborated the coordinated upregulation of G6PD and SPP1 at the protein level in an independent, treatment-naïve surgical cohort (n = 114). Together, our findings nominate a coordinated metabolically active and SPP1-enriched microenvironmental state that is associated with aggressive tumor biology and may provide a biological rationale for future studies of locoregional-systemic therapy resistance in HCC.

## Materials and methods

### Patient specimens and surgical cohort

This study utilized fresh liver specimens for the exploratory single-cell discovery phase and an independent retrospective cohort for clinical validation. For the scRNA-seq analysis, fresh liver tissues were collected from five patients between July 2024 and October 2024. These included one therapy-progressive HCC lesion, two treatment-naïve HCC tumors, and two adjacent non-tumor liver tissues, all obtained via surgical resection or biopsy.

For protein-level clinical validation, we enrolled a retrospective surgical cohort of 114 treatment-naïve HCC patients from the Fourth Hospital of Hebei Medical University (East Campus) who underwent hepatectomy between January 2019 and December 2022. Inclusion criteria were: (Bray et al., 2024) postoperative pathological confirmation of HCC; (Llovet et al., 2022) no prior anticancer therapy before hepatectomy; and (Cheng et al., 2022) availability of formalin-fixed, paraffin-embedded (FFPE) tumor tissue blocks. Exclusion criteria were: (Bray et al., 2024) loss to follow-up and (Llovet et al., 2022) incomplete clinical data. Paired adjacent non-tumor liver tissues were available for 39 patients. Baseline clinicopathological characteristics, including alpha-fetoprotein (AFP), microvascular invasion (MVI), Barcelona Clinic Liver Cancer (BCLC) stage and tumor burden score (TBS), were recorded. Patients were followed until November 25, 2025. Follow-up time was calculated from the date of surgery to the date of last contact or death. The median potential follow-up time was 63.1 months, as estimated using the reverse Kaplan-Meier method.

All patients involved in the fresh tissue collection provided written informed consent. The entire study was conducted in accordance with the Declaration of Helsinki and approved by the Ethics Committee of the Fourth Hospital of Hebei Medical University (Approval No. 2024KS123).

### Single-cell RNA sequencing processing and annotation

Raw single-cell sequencing data were demultiplexed and mapped to the human reference genome to generate gene-barcode count matrices. Downstream quality control and analysis were performed utilizing the Seurat package (v.5.1.0) in R (v.4.3.2). To ensure data fidelity, rigorous filtering criteria were applied: cells expressing fewer than 300 or greater than 5,000 genes, or exhibiting a mitochondrial gene expression fraction exceeding 25%, were excluded. Putative computational doublets were identified and removed using the scDblFinder package (v.1.20.0). Following log-normalization and selection of the top 2,000 highly variable genes, principal component analysis (PCA) was conducted. Sample-specific batch effects in fresh liver specimens were adjusted using the Harmony package, and Uniform Manifold Approximation and Projection (UMAP) was conducted on the top 20 principal components. Unsupervised graph-based clustering was executed at a resolution of 0.7. Cell clusters were definitively annotated into 12 major populations based on canonical marker genes curated from the CellMarker 2.0 database (http://bio-bigdata.hrbmu.edu.cn/CellMarker/) (Hu et al., 2023) via the easybio package (v.1.1.1). Because the therapy-progressive group comprised a single donor, treatment status is inherently confounded with donor identity. Accordingly, differential expression and cell–cell communication analyses reflect cell-level inference within this index case and cannot formally distinguish therapy effects from inter-patient variation.

### Hepatocyte-specific differential expression and pathway analysis

To reduce confounding from mixed cellular composition and focus on tumor-intrinsic signals, differential expression analysis was strictly restricted to the annotated hepatocyte compartment. Differentially expressed genes (DEGs) between hepatocytes from the therapy-progressive lesion and those from treatment-naïve tissues were determined using the Wilcoxon rank-sum test. While these cell-level statistical comparisons served as an initial screen, potential pseudoreplication bias was mitigated by downstream validation in external bulk and clinical patient cohorts. Significance was established at an absolute average log2 fold-change (|avg_log2FC|) > 0.25 and a Benjamini-Hochberg (BH) false discovery rate (FDR) adjusted P-value < 0.05. To decode the functional phenotypic shifts across different cell types, we queried cellular pathway variations utilizing the ReactomeGSA package (v.1.16.1), quantifying the top distinctive biological pathways remodeling the cell states.

### Transcriptomic data acquisition and lactylation-related gene curation

Bulk RNA-seq data together with clinical annotations were obtained from the TCGA-LIHC cohort, which includes 371 tumor samples and 50 normal controls, with 361 samples having complete survival data. This data was obtained using the TCGAbiolinks package (v.2.35.3) (Colaprico et al., 2016). External validation was conducted with 240 RNA-seq liver cancer cases from the ICGC-LIRI-JP cohort, comprising 240 tumor samples and 197 normal controls (Zhang J. et al., 2019). As a candidate-prioritization filter, a set of 462 lactylation-related genes was curated (note: this list was used solely for gene screening; lactylation was not experimentally measured). This curation integrated the “Lactylation” module from the GeneCards database (https://www.genecards.org/) (Safran et al., 2010) with experimentally validated genes from contemporary literature (Sun et al., 2024). For the TCGA-LIHC cohort, bulk-level DEGs between tumor and non-tumor adjacent tissues were identified using the DESeq2 package (v.1.46.0). Conservative thresholds of |log2FC| > 0.5 and a BH-FDR adjusted P-value < 0.05 were applied.

### Prognostic signature construction

Candidate genes were identified by intersecting three gene sets: (i) the curated lactylation-related gene set; (ii) index-case hepatocyte DEGs from scRNA-seq; and (iii) bulk-level tumor DEGs from TCGA-LIHC. Functional enrichment of these candidate genes in Gene Ontology (GO) terms and Kyoto Encyclopedia of Genes and Genomes (KEGG) pathways was evaluated with the clusterProfiler package (v4.15.1).

For prognostic model development, univariable Cox regression analysis was first applied to candidate genes in the TCGA-LIHC cohort to identify survival-associated features. Subsequently, LASSO penalized Cox regression was performed to minimize model overfitting and derive a parsimonious signature. The prognostic risk score was calculated by summing the products of each selected gene’s normalized expression value and its corresponding LASSO coefficient. Patients from both the TCGA-LIHC cohort and the independent ICGC LIRI-JP validation cohort were divided into high- and low-risk groups according to the cohort-specific median risk score. The prognostic value of this stratification was assessed by Kaplan-Meier survival analysis with log-rank testing.

### Immune microenvironment deconvolution and drug sensitivity inference

To characterize the immune microenvironmental features associated with the risk signature, the relative infiltration abundances of 22 distinct immune subsets in the TCGA-LIHC cohort were deconvoluted using the CIBERSORT algorithm via the IOBR package (v.2.34.0). The functional activation cascades of anticancer immunity were mapped by quantifying the seven-step Cancer Immunity Cycle through the Tracking Tumor Immunophenotype (TIP) framework (http://biocc.hrbmu.edu.cn/TIP/) (Xu et al., 2018). As an exploratory analysis, the half-maximal inhibitory concentrations (IC50) for standard systemic agents—including tyrosine kinase inhibitors (e.g., lenvatinib, sorafenib) and chemotherapeutics (e.g., oxaliplatin, fluorouracil)—were predicted utilizing the oncoPredict package (v.1.2), calibrated against the Cancer Therapeutics Response Portal (CTRP2) training reference (https://portals.broadinstitute.org/ctrp/).

### Single-cell signature mapping and intercellular communication

To bridge macro-level bulk prognostic stratification with single-cell functional states, the activity of the multiparametric signature was quantified within individual cells using the AUCell package (v.1.28.0). Cells were systematically classified into signature-high and signature-low states based on the threshold derived from AUCell. To explore intercellular communication patterns associated with this transcriptional state, intercellular communication patterns and exact ligand-receptor modalities (emphasizing SPP1 signaling pathways) were constructed and compared between the signature states, as well as between therapy-progressive and treatment-naïve specimens, employing the CellChat package (v.2.1.2).

### Immunohistochemistry and semi-quantitative evaluation

Following deparaffinization and rehydration of 4-μm formalin-fixed, paraffin-embedded (FFPE) sections from HCC tumors and adjacent non-tumor liver specimens, endogenous peroxidase activity was quenched using 3% hydrogen peroxide. After EDTA-based antigen retrieval, consecutive serial sections derived from the same FFPE tissue block of each patient were prepared for paired G6PD and SPP1 staining. These serial sections were incubated separately overnight at 4 °C with anti-G6PD antibody (Proteintech, 25413-1-AP, 1:300) or anti-SPP1 antibody (Proteintech, 22952-1-AP, 1:300), respectively. Following incubation with an HRP-conjugated secondary antibody, signals were visualized using DAB. Sections were then counterstained with hematoxylin, dehydrated, cleared, and mounted.

Immunohistochemistry images were acquired using an Olympus UC90 microscope camera. For each tissue section, five representative non-overlapping fields of view at ×20 magnification were randomly selected. Protein expression levels of G6PD and SPP1 were semi-quantitatively assessed using ImageJ software (NIH, USA) with the IHC Profiler plugin. After background correction with the Rolling Ball algorithm (Radius = 100), the H-score was calculated automatically. The formula utilized was: H-score = 3 × (% of strongly positive cells) + 2 × (% of moderately positive cells) + 1 × (% of weakly positive cells), resulting in a score ranging from 0 to 300. The final H-score for each patient was calculated as the mean value of the five independent fields. All H-score results were verified by an experienced pathologist who was blinded to the clinical data.

### Statistical analysis

Statistical analyses were conducted primarily using R (version 4.3.2). Baseline clinicopathological comparisons (Supplementary Table S6) were performed using GraphPad Prism (version 9.5.1), which was also used for visualization of IHC comparisons. Optimal cutoff determination was performed using X-tile (version 3.6.1; Yale University). Continuous variables are presented as mean ± standard deviation or median (interquartile range, IQR). Depending on data normality and homogeneity of variance, inter-group comparisons were performed using Student’s t-test (independent samples or Welch’s correction) or the Mann-Whitney U test. Paired samples were evaluated via the Wilcoxon signed-rank test. Categorical variables are reported as counts (percentages) and were compared using Pearson’s χ^2^ or Fisher’s exact test. Recurrence-free survival (RFS) and overall survival (OS) served as the primary and secondary endpoints, respectively. The optimal cutoff for stratifying G6PD expression was determined via X-tile. Survival curves were estimated using the Kaplan-Meier method and compared by the log-rank test. Univariable Cox and multivariable Cox proportional hazards models were employed to estimate hazard ratios (HRs) and identify independent prognostic factors. Furthermore, restricted cubic splines (RCS) with three knots (rms R package) were used to model potential nonlinear associations between the continuous G6PD H-score and the risk of recurrence. Correlations were assessed using Spearman’s rank correlation. All statistical tests were two-sided, and a P < 0.05 was considered statistically significant.

## Results

### Exploratory single-cell profiling identifies microenvironmental remodeling in therapy-progressive HCC

**FIGURE 1:**
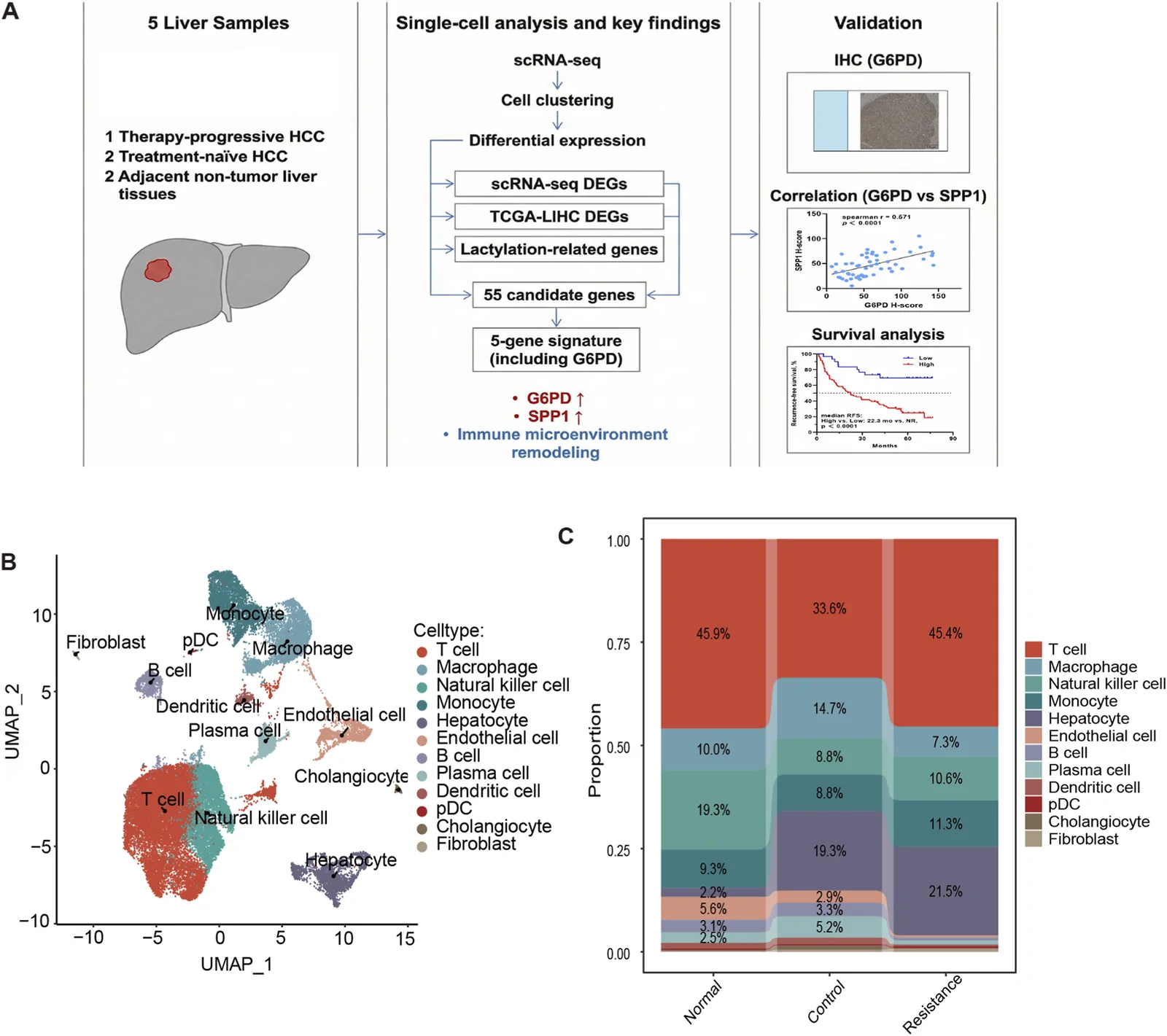
Single-cell transcriptomic landscape of therapy-progressive and treatment-naïve hepatocellular carcinoma (HCC). **(A)** Study design showing five liver specimens, including one therapy-progressive tumor, two treatment-naïve tumors, and two adjacent non-tumor tissues. **(B)** Uniform manifold approximation and projection (UMAP) visualization showing 12 distinct clusters representing major cell types. **(C)** Relative proportions of major cell types across sample groups, highlighting compositional differences.

We performed exploratory scRNA-seq on five liver specimens, including one therapy-progressive HCC lesion, two treatment-naïve HCC tumors, and two adjacent non-tumor liver samples (non-paired) (Figure 1A). The therapy-progressive (resistance) lesion was obtained from a BCLC stage B patient treated with HAIC (FOLFOX) plus lenvatinib and sintilimab, in whom radiographic assessment demonstrated disease progression from stable disease (SD) to progressive disease (PD) by mRECIST criteria after 3 cycles of treatment.

After quality control (percent.mt < 25%, nFeature_RNA 300–5,000) and doublet removal, we retained 32,950 high-quality cells for downstream analysis. We integrated datasets using Seurat and mitigated batch effects using Harmony, followed by PCA-based clustering (top 20 PCs; resolution = 0.7). This analysis identified 12 major cell types, including hepatocytes, cholangiocytes, endothelial cells, T cells, NK cells, monocytes, macrophages, B cells, plasma cells, dendritic cells, plasmacytoid dendritic cells (pDCs), and fibroblasts (Figure 1B). These cluster annotations were verified using known marker genes (Supplementary Figure S1).

Cell-type composition differed across groups, as the therapy-progressive tumor displayed a distinct cellular distribution compared with treatment-naïve tumors and non-tumor liver (Figure 1C), reflecting microenvironmental remodeling during therapy. While the discovery cohort is limited to a single therapy-progressive lesion (n = 1), this single-cell profiling served strictly to generate exploratory hypotheses, which were subsequently validated in independent, large-scale cohorts to ensure clinical generalizability.

### Hepatocyte-specific analysis identifies candidate programs in an index therapy-progressive lesion

**FIGURE 2:**
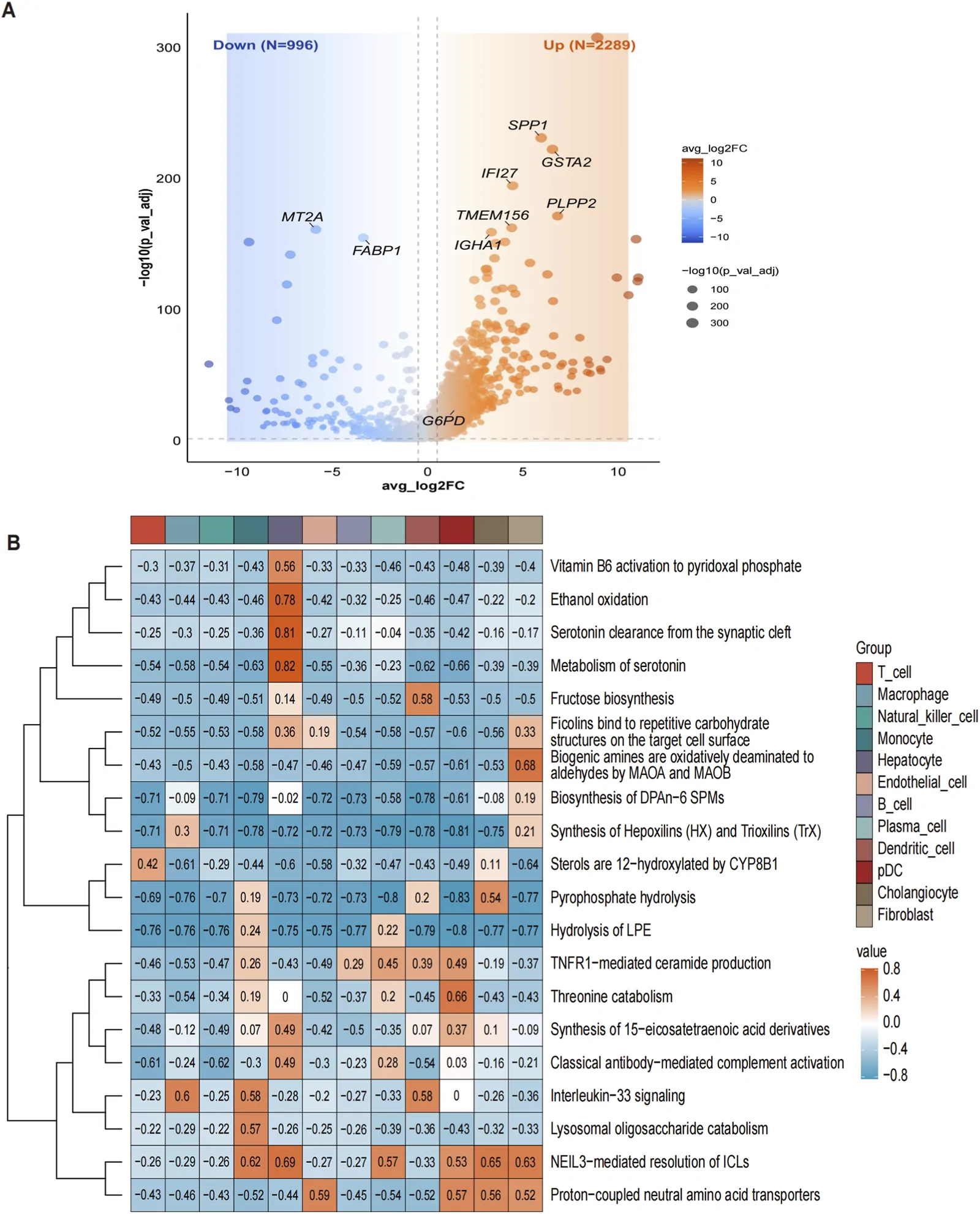
Hepatocyte-intrinsic transcriptional alterations in therapy-progressive and treatment-naïve hepatocellular carcinoma. **(A)** Volcano plot of differentially expressed genes in hepatocytes comparing the index therapy-progressive lesion and treatment-naïve tumors. Key genes including SPP1 and G6PD are highlighted. **(B)** Pathway enrichment analysis across major cell types, showing hepatocyte-specific enrichment of metabolic and DNA repair pathways.

To limit confounding from mixed cellular composition, we restricted the analysis to tumor hepatocytes. Comparing hepatocytes from therapy-progressive and treatment-naïve lesions revealed 4,552 differentially expressed genes (DEGs) (|avg_log2FC| > 0.25, adjust P value < 0.05) (Figure 2A; Supplementary Table S1). Among the upregulated genes, we observed increased expression of SPP1, GSTA2, MT2A, and FABP1. Furthermore, G6PD was markedly elevated in hepatocytes from the index therapy-progressive lesion (Figure 2A), suggesting a potential role of the pentose phosphate pathway (PPP) in this tumor state.

Pathway analysis across annotated cell types showed compartment-specific programs: hepatocytes were enriched for ethanol oxidation, serotonin clearance/metabolism, vitamin B6 activation to pyridoxal phosphate, and NEIL3-mediated resolution of interstrand crosslinks (Figure 2B). These pathways point to metabolic reprogramming, microenvironmental signal modulation, and DNA damage repair (Mishra et al., 2025; Aliyaskarova et al., 2023; Zhang et al., 2025)-mechanisms known to drive therapy resistance and overall tumor progression.

### Integrative transcriptomic analysis yields a metabolic-transcriptional prognostic signature

**FIGURE 3:**
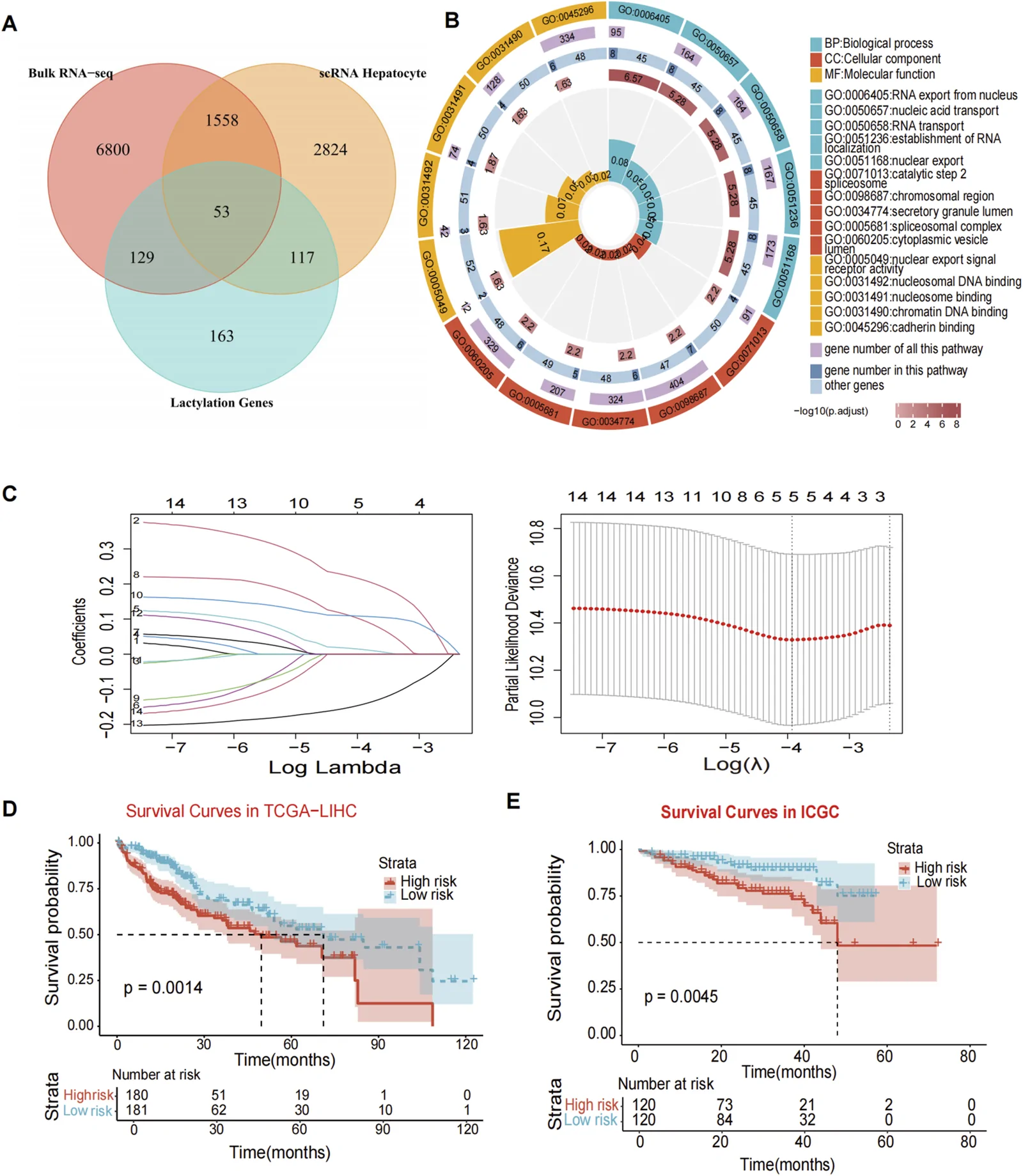
Identification of lactylation-related candidates and construction of a prognostic signature. **(A)** Venn diagram showing overlap among lactylation-related genes, single-cell RNA sequencing differentially expressed genes (DEGs), and TCGA-LIHC bulk DEGs. **(B)** Functional enrichment analysis of overlapping genes, highlighting RNA processing, chromatin regulation, and metabolic pathways. **(C)** Least absolute shrinkage and selection operator (LASSO) Cox regression for feature selection of prognostic genes. **(D)** Kaplan–Meier survival analysis in TCGA-LIHC cohort stratified by risk score. **(E)** External validation of the risk signature in the ICGC cohort.

Because lactate-linked post-translational modifications such as lysine lactylation have been proposed as interfaces between metabolic states and gene regulation (Cai et al., 2025; Sun et al., 2025), we adopted a lactylation-related gene framework to prioritize candidates with translational potential. A total of 462 unique lactylation-related genes were curated for downstream analysis (Supplementary Table S2). To identify robust candidates supported across modalities, we intersected this lactylation-related gene list with tumor-hepatocyte DEGs from scRNA-seq (n = 4,552) and tumor-versus-normal DEGs from TCGA-LIHC bulk RNA-seq (n = 8,540) (Figure 3A). This approach yielded 53 overlapping genes (Supplementary Table S3).

Functional enrichment analysis of lactylation-related differentially expressed genes revealed significant enrichment in RNA transport/nuclear export, spliceosome- and chromosome-related components, and chromatin/nucleosome binding functions (Figure 3B). Together, these results indicate that lactylation-related differentially expressed genes are predominantly associated with post-transcriptional RNA processing, chromosomal structural regulation, and metabolic processes. These functional categories are associated with post-transcriptional RNA processing, chromatin regulation, and carbohydrate and amino acid metabolism, providing a rationale for prioritizing these genes as prognostic candidates rather than implying a specific regulatory mechanism in HCC.

We then performed prognostic triaging in TCGA-LIHC. Univariable Cox regression identified 14 genes significantly associated with survival (Supplementary Table S4), from which LASSO Cox regression derived a parsimonious five-gene signature comprising BZW1, RAN, NR6A1, G6PD, and APOC2 (Figure 3C). The risk score was calculated as follows: 0.213 × BZW1 + 0.013 × RAN + 0.098 × NR6A1 + 0.108 × G6PD − 0.144 × APOC2. Using the median risk score as the cutoff, the five-gene signature significantly stratified overall survival in TCGA-LIHC (Figure 3D; P < 0.05) and in the external validation cohort ICGC LIRI-JP (Figure 3E; P < 0.05). We carried this risk-based stratification forward to examine downstream differences in the immune microenvironment and therapeutic vulnerability between patient subgroups.

### The five-gene risk signature is associated with distinct immune microenvironment features and predicted drug sensitivity profiles in bulk HCC cohorts

**FIGURE 4:**
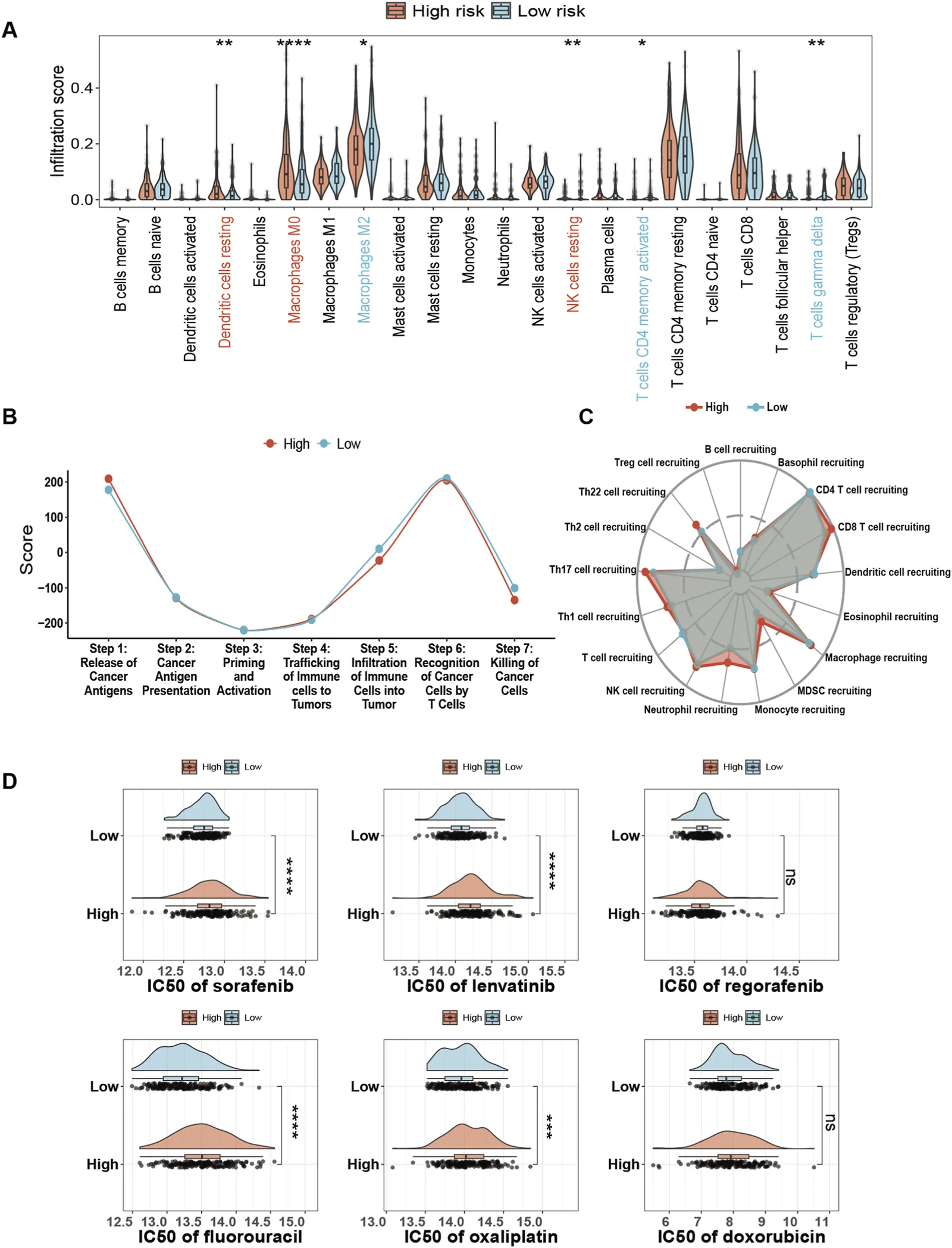
Immune landscape and therapeutic response associated with the risk signature. **(A)** Immune cell infiltration profiles estimated by CIBERSORT (Cell-type Identification by Estimating Relative Subsets of RNA Transcripts) across risk groups. **(B)** Cancer immunity cycle activity comparison between high- and low-risk groups. **(C)** Immune cell recruitment signatures across risk groups. **(D)** Predicted drug sensitivity (IC50, half-maximal inhibitory concentration) differences between risk groups.

To characterize the tumor microenvironment context associated with the risk score, we performed immune deconvolution and immune-function profiling in TCGA-LIHC. Using CIBERSORT (LM22), we estimated the abundance of 22 immune cell subsets and compared infiltration between the high- and low-risk groups. High-risk tumors exhibited significantly different immune infiltration patterns, including higher Dendritic cells resting, Macrophages M0, and NK cells resting and lower Macrophages M2, T cells CD4 memory activated, and T cells gamma delta relative to low-risk tumors (Figure 4A).

We next quantified the cancer immunity cycle across risk groups. The high-risk group showed greater activity in cancer antigen release (step 1), whereas the low-risk group showed greater activity in immune cell infiltration into tumors (step 5) and cancer cell killing (step 7) (Figure 4B). In addition, several immune cell–recruitment signatures—including CD8 T cell recruiting, macrophage recruiting, neutrophil recruiting, and Th17 cell recruiting—were broadly elevated in the high-risk group (Figure 4C). Multiple immune checkpoint genes (e.g., PDCD1/PD-1, CD274/PD-L1, CTLA4, HAVCR2/TIM-3, and TIGIT) were significantly upregulated in the high-risk group, characterizing an immunosuppressive microenvironment where enhanced checkpoint signaling co-occurs with features of immune evasion (Supplementary Figure S2A). To determine if the risk signature correlates with drug response, we inferred drug sensitivity using oncoPredict (trained on CTRP2) and compared predicted IC50 values between risk groups. The high-risk group showed significantly higher predicted IC50 values (reflecting lower sensitivity) for sorafenib, lenvatinib, fluorouracil, and oxaliplatin, while no significant differences were observed for regorafenib or doxorubicin (Figure 4D). Notably, G6PD expression correlated positively with the predicted IC50 of lenvatinib, fluorouracil, and oxaliplatin, suggesting a potential association between G6PD and reduced drug sensitivity (Supplementary Figure S2B). As these oncoPredict estimates are derived from cell-line pharmacogenomic data (CTRP2) rather than clinical outcomes, they should be regarded as exploratory and do not directly reflect clinical response.

### Mapping the signature back to scRNA-seq identifies a signature-high cell state and enhanced SPP1-linked communication

**FIGURE 5:**
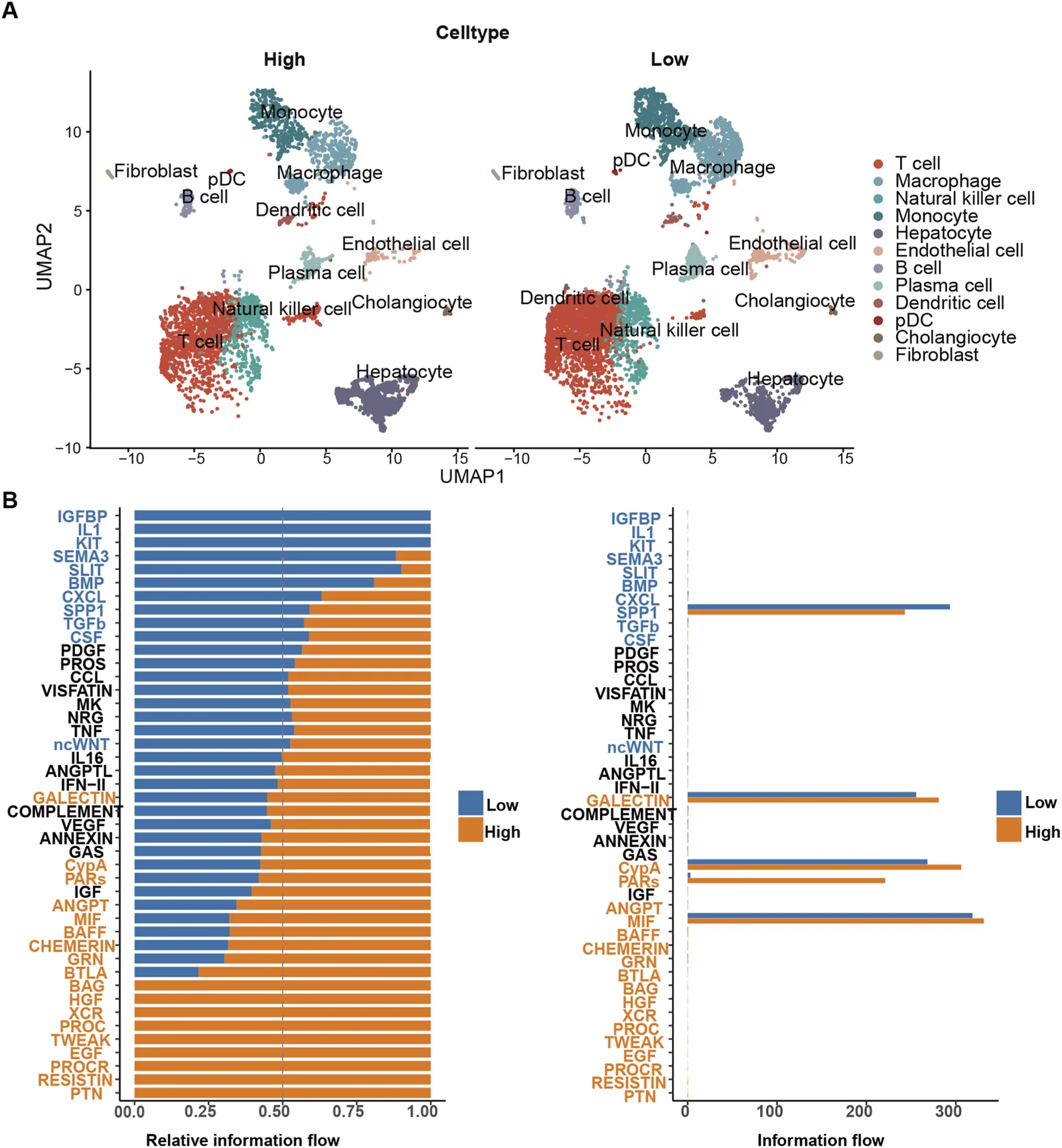
Single-cell mapping of the risk signature and intercellular communication analysis. **(A)** UMAP showing distribution of signature-high and signature-low cells. **(B)** CellChat-derived information flow of key pathways in signature-high versus signature-low groups.

To connect bulk-level risk stratification to cell-resolved biology, we quantified signature activity at single-cell resolution using AUCell. Cells were classified into signature-high and signature-low states based on an empirically determined AUCell threshold (Supplementary Figure S3A). The signature-high state displayed a distinct distribution in UMAP space (Figure 5A) and was enriched in hepatocytes and NK cells (Supplementary Figure S3B), indicating that the bulk risk signature reflects an underlying cell-state program observable in scRNA-seq.

We then exploratorily assessed differences in intercellular communication between signature-high and signature-low states using CellChat. Signature-high tumors exhibited greater overall communication strength and information flow than signature-low tumors (Figure 5B; Supplementary Figure S3C). Pathway-level analysis showed that the signature-high state was dominated by signaling networks such as GALECTIN and MIF, which typically reflect a generalized, highly aggressive tumor-intrinsic phenotype. Interestingly, SPP1 signaling information flow was higher in the signature-low compartment than in the signature-high compartment (Figure 5B; Supplementary Figure S3D). This likely reflects the cellular composition of the signature-low state, which is enriched in immune and stromal populations, including macrophages and fibroblasts, that are major senders and receivers of SPP1 signaling. Thus, this pattern does not contradict the G6PD–SPP1 association observed in tumor hepatocytes. To further assess SPP1 dynamics in therapy resistance independently of the AUCell-based classification, we performed a specimen-level CellChat analysis comparing the therapy-progressive tumor with treatment-naïve controls. This analysis showed that hepatocytes from the therapy-progressive lesion, and to a lesser extent cholangiocytes, were the main sources of SPP1 signaling to immune and stromal compartments (Supplementary Figure S3E; Supplementary Table S5). These findings are consistent with the hepatocyte DEG analysis, which identified SPP1 as a key upregulated gene in the therapy-progressive lesion.

### Clinical validation demonstrates that G6PD overexpression is associated with adverse recurrence-free survival and correlates with SPP1

**FIGURE 6:**
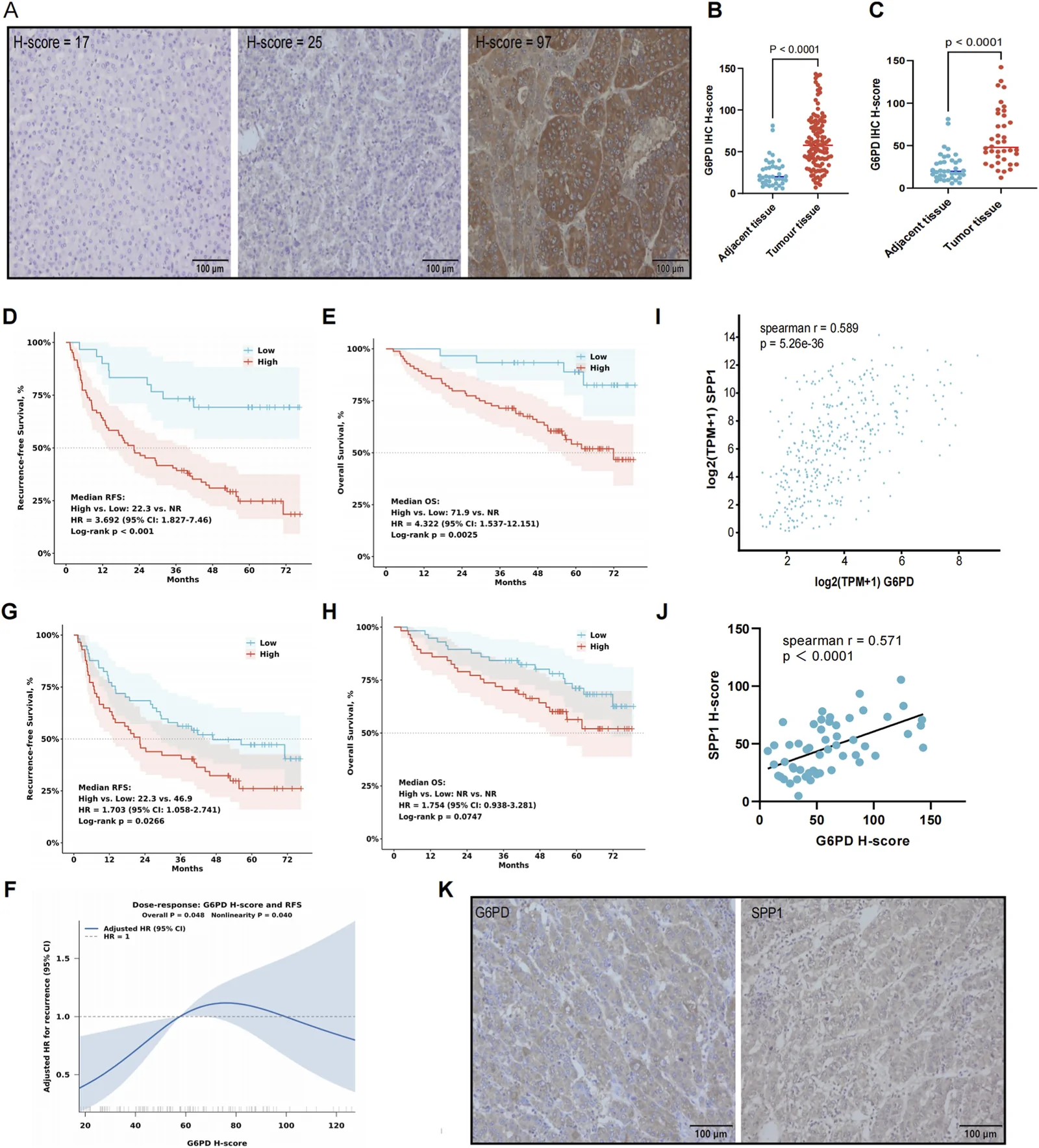
G6PD expression predicts recurrence and correlates with SPP1 in hepatocellular carcinoma. **(A)** Representative immunohistochemical staining of G6PD in adjacent non-tumor liver tissue (left), tumor tissue with low G6PD expression (middle), and tumor tissue with high G6PD expression (right). **(B,C)** Comparison of G6PD H-scores between tumor and non-tumor tissues (unpaired and paired). **(D,E)** Kaplan–Meier curves for recurrence-free survival (RFS) and overall survival (OS) based on exploratory X-tile-defined G6PD expression groups. **(F)** Restricted cubic spline analysis of the association between G6PD H-score and recurrence risk. **(G,H)** Kaplan–Meier curves for RFS and OS based on median G6PD H-score stratification. **(I,J)** Correlation between G6PD and SPP1 expression at mRNA and protein (immunohistochemistry) levels. **(K)** Representative immunohistochemical staining of G6PD and SPP1 from the same patient specimen, demonstrating concordant staining intensity patterns. These images are presented as a qualitative illustration and do not imply spatial co-localization.

Based on the computational identification of G6PD as a candidate gene in HCC, we next evaluated its protein expression in an independent surgical cohort. We assessed G6PD expression by immunohistochemistry in 114 treatment-naïve HCC tumors and 39 adjacent non-tumor liver tissues, quantifying it via the H-score (range 0–300). Representative IHC images showed weak staining in adjacent liver tissues and variable cytoplasmic staining in tumor tissues (Figure 6A). Tumor tissues exhibited significantly higher G6PD H-scores than adjacent non-tumor liver in both unpaired and paired comparisons (Figures 6B,C; both P < 0.0001).

To prioritize a statistically rigorous approach, we first analyzed G6PD H-score as a continuous variable. In univariable Cox regression, higher H-score was significantly associated with shorter RFS (HR = 1.086 per 10-point increase, 95% CI 1.015–1.162, P = 0.017). After multivariable adjustment for AFP, BCLC stage, and microvascular invasion, the association remained directionally consistent, though it attenuated to non-significance (HR = 1.069 per 10-point increase, 95% CI 0.998–1.146, P = 0.058), and the bootstrap-corrected 95% CI (0.990–1.194) encompassed the null, likely reflecting the limited statistical power of this cohort. Importantly, a sensitivity analysis substituting tumor burden score for BCLC stage yielded a significant and consistent result (HR = 1.087, 95% CI 1.014–1.166, P = 0.020), supporting a consistent trend for this association. Model diagnostics confirmed the proportional hazards assumption (global P = 0.327), absence of collinearity (all VIF < 1.2), and good internal validity (bootstrap-corrected C-index = 0.671, optimism = 0.014). RCS analysis revealed a nonlinear positive association between H-score and recurrence risk (overall P = 0.048, nonlinearity P = 0.040; Figure 6F), with a more pronounced risk elevation at higher expression levels.

For clinical stratification, X-tile analysis identified an exploratory H-score cutoff of 40.3 (Miller–Siegmund P = 0.0038), classifying 84 patients as G6PD-high and 30 as G6PD-low. The G6PD-high group had a substantially higher recurrence rate (72.6% vs. 30.0%, P < 0.0001; Supplementary Table S6). Kaplan-Meier analysis demonstrated significantly shorter RFS (median 22.3 months vs. not reached; Figure 6D; log-rank P < 0.001) and shorter OS (median 71.9 months vs. not reached; Figure 6E; log-rank P = 0.0025) in the G6PD-high group. Multivariable Cox regression confirmed G6PD-high status as an independent predictor of shorter RFS (HR = 3.503, 95% CI 1.696–7.237, P < 0.001; Table 1). As this cutoff was derived and evaluated within the same cohort, these results should be considered exploratory and require independent validation. Notably, no significant difference in post-recurrence treatment distribution was observed between groups (Fisher’s exact test, P = 0.891; Supplementary Table S6), suggesting that the survival difference likely reflects the underlying tumor biology, although potential confounding from subsequent clinical management cannot be completely ruled out.

**TABLE 1:** . Multivariable Cox regression analysis for recurrence-free survival.

Variable	HR	95% CI for HR	P Value
Serum AFP level, ng/mL (≥400 vs. <400)	2.276	1.381–3.751	0.0012
BCLC stage (B&C vs. 0&A)	3.281	1.781–6.043	0.0001
Microvascular invasion (Present vs. Absent)	1.498	0.868–2.588	0.1469
G6PD H-score (per 10-point increase)	1.069	0.998–1.146	0.0583

G6PD H-score was entered as a continuous variable. The adjusted association did not reach statistical significance, and the bootstrap-corrected 95% CI (0.990–1.194) included the null. A sensitivity analysis substituting tumor burden score for BCLC stage yielded a significant and consistent result (HR, 1.087, 95% CI, 1.014–1.166, P = 0.020). The proportional hazards assumption was satisfied for this model (global P = 0.327). Bootstrap-corrected C-index = 0.671 (apparent 0.685, optimism 0.014). Abbreviations: HR, hazard ratio; CI, confidence interval; AFP, alpha-fetoprotein; BCLC, barcelona clinic liver cancer; G6PD, glucose-6-phosphate dehydrogenase.

Using the cohort median H-score (57.7) as a data-independent threshold, the high-expression group showed shorter RFS (median 22.3 vs. 46.9 months; Figure 6G; log-rank P = 0.027) and a non-significant trend toward shorter OS (Figure 6H; log-rank P = 0.075). Univariable analysis supported an association with recurrence risk (HR = 1.703, 95% CI 1.058–2.741, P = 0.028), though significance was not maintained after multivariable adjustment (HR = 1.589, 95% CI 0.964–2.618, P = 0.069). Across all three analytical approaches, higher G6PD expression consistently correlated with increased recurrence risk, with the X-tile threshold providing the most effective stratification in this exploratory analysis.

Because our scRNA-seq analysis identified G6PD and SPP1 as co-upregulated genes in hepatocytes from the index therapy-progressive lesion, we next evaluated their association at the tissue level. Using the GEPIA3 database (Kang et al., 2025), we found that G6PD and SPP1 mRNA levels were significantly correlated in the bulk TCGA-LIHC cohort (n = 371; Spearman r = 0.589, P = 5.26 × 10^−36^). We validated this association at the protein level by tumor IHC in a subset of 54 patients from our surgical cohort. Consistent with the transcriptomic data, G6PD and SPP1 protein H-scores showed a significant positive correlation (Spearman r = 0.571, P < 0.0001) (Figures 6I,J). To provide an intuitive within-patient illustration, we additionally present representative staining from the same tumor specimen, showing concordant staining intensity patterns of G6PD and SPP1 (Figure 6K). Although these images do not demonstrate direct spatial co-localization, they qualitatively support the tissue-level concordance observed across the cohort. Consistent with our single-cell data, these findings highlight an association between G6PD expression and SPP1-related signaling. While the exact mechanisms require further study, our results suggest that metabolic adaptation and microenvironmental remodeling are closely connected in HCC.

## Discussion

This study characterizes an aggressive and immunosuppressive tumor state in hepatocellular carcinoma associated with coordinated upregulation of G6PD-driven pentose phosphate pathway activity and SPP1-related intercellular signaling. We integrated single-cell profiling of an index therapy-progressive lesion with cross-cohort survival modeling and independent protein-level validation to derive a five-gene lactylation-related prognostic signature (BZW1, RAN, NR6A1, G6PD, APOC2). This signature stratified overall survival in two independent cohorts and identified an immune-inflamed but functionally suppressed microenvironment with reduced predicted sensitivity to frontline systemic agents. Together, these results provide a biological framework linking intracellular metabolic reprogramming to immune evasion and therapeutic failure in HCC.

G6PD upregulation in therapy-progressive hepatocytes aligns with the established role of PPP hyperactivation in maintaining NADPH-dependent antioxidant defenses and supporting nucleotide biosynthesis under genotoxic and oxidative stress (Patra and Hay, 2014). In HCC, an epigenetic METTL3-TRIM21-G6PD axis has been reported to confer oxaliplatin resistance, providing mechanistic support for PPP-mediated tolerance to HAIC-based regimens (Jin et al., 2025). In addition, inhibition of G6PD-driven redox homeostasis enhances chemosensitivity in gastrointestinal cancers (Ju et al., 2017). Pharmacological studies with the selective inhibitor G6PDi-1 showed that disrupting PPP-mediated redox balance compromised oxidative stress tolerance and colony formation in cancer cell models and selectively suppressed proinflammatory cytokine production by T cells and the oxidative burst in neutrophils (Ghergurovich et al., 2020), underscoring both metabolic and immunological significance. Clinically, elevated tumor G6PD expression has been associated with metastatic propensity and worse survival outcomes in treatment-naïve HCC and has been linked to earlier recurrence and shorter disease-free survival in other tumor types such as breast cancer (Lu et al., 2018; Mele et al., 2019). Validating this clinical relevance, our independent 114-patient cohort demonstrated that G6PD overexpression was associated with shorter recurrence-free survival—with independent prognostic significance supported by exploratory X-tile–based stratification—and revealed a non-linear positive association between G6PD H-score and recurrence risk by restricted cubic spline analysis (overall P = 0.048, nonlinearity P = 0.040). Integrating this prognostic baseline with our single-cell data, we propose that G6PD-associated metabolic reprogramming may not merely reflect a transient therapy-induced response, but may instead mark an intrinsically aggressive tumor state associated with metastatic potential and treatment refractoriness. Its predictive value for ICI-based combinations warrants future prospective validation.

Because lysine lactylation has been proposed as an interface between metabolism and gene regulation (Zhang et al., 2026), we used a curated lactylation-related gene list purely as a computational candidate-screening filter. This screen identified a five-gene signature that reflects multiple biological processes relevant to tumor progression. These include translational regulation and glycolytic programs (BZW1) (Li et al., 2022), chromosomal instability (RAN) (El-Tanani et al., 2023), metabolism-associated nuclear receptor signaling (NR6A1) (Liu et al., 2025), PPP-related redox adaptation (G6PD) (Patra and Hay, 2014), and apolipoprotein C-II (APOC2). We did not measure lactate levels or lysine lactylation and make no claim that the five nominated genes are lactylation-regulated; although our study infers lactylation-associated biology from transcriptomic data, this correlative approach offers a starting point for future experimental validation. The consistent prognostic stratification achieved by this signature across both the TCGA-LIHC and ICGC LIRI-JP cohorts confirms its applicability across different populations. Future prospective studies in patients receiving ICI-based combination therapy are warranted to confirm its predictive clinical utility.

The high-risk signature mapped onto a tumor microenvironment with increased immune-recruitment signals and upregulation of multiple immune checkpoints (PD-1, PD-L1, TIM-3, CTLA-4, and TIGIT), but reduced activity in the effector phases of the cancer–immunity cycle, including T-cell infiltration and cytotoxic killing. This “inflamed-but-ineffective” phenotype matches the immune-specific HCC subclass described by Sia et al. (2017) and is supported by single-cell and high-dimensional profiling studies that demonstrated pervasive T-cell exhaustion and multi-checkpoint coexpression in advanced (Zhang et al., 2019; Li et al., 2024b; Ma et al., 2019). The concurrent upregulation of multiple checkpoint molecules suggests that resistance to single-agent immunotherapy may be driven by layered immune suppression rather than immune absence, supporting the rationale for combining checkpoint blockade with metabolic or TME-targeting strategies. In silico drug-response inference suggested reduced sensitivity to sorafenib, lenvatinib, fluorouracil, and oxaliplatin in high-risk tumors; these exploratory computational inferences are hypothesis-generating and require prospective validation.

Recent high-resolution transcriptomic and multi-omics studies of the HCC microenvironment have predominantly highlighted the stromal and immune compartments-particularly SPP1^+^tumor-associated macrophages and cancer-associated fibroblasts-as the primary drivers of immune exclusion and therapy resistance (Zhang Q. et al., 2019; Liu et al., 2023; Eun et al., 2023). While these host populations are indisputably central to microenvironmental immunosuppression, our single-cell mapping suggests a potential division of labor between tumor and stromal compartments. Interestingly, although the broader SPP1 intercellular signaling network appeared predominantly enriched within the signature-low compartment (comprising diverse immune and stromal receivers), our orthogonal analysis of the index therapy-progressive lesion identified hepatocytes as among the main sources of outgoing SPP1 signaling toward immune and stromal compartments (Supplementary Figure S3E). This suggests a potential paracrine interaction in which G6PD-active tumor hepatocytes may contribute to SPP1-mediated signaling toward stromal and immune constituents. This is consistent with recent spatial transcriptomic evidence showing that tumor-derived SPP1 can induce localized fibroblast chemotaxis and activation (Tong et al., 2024). The consistent concurrent expression of G6PD and SPP1-validated at both the transcriptomic and protein levels in our clinical cohorts-suggests a coordinated upregulation pattern worthy of further investigation. While the exact mechanisms require further study, this parallel upregulation suggests a potential association between intracellular metabolic reprogramming linked to G6PD and extracellular microenvironmental remodeling related to SPP1. However, whether these processes functionally cooperate under therapeutic pressure to sustain treatment refractoriness requires further experimental validation.

This study has several key limitations. First, the single-cell discovery phase was restricted to an index therapy-progressive lesion from a single patient. As a consequence, differential-expression and cell–cell communication P values reflect cell-level pseudoreplication, and the observed differences cannot be formally attributed to treatment effects rather than donor-specific variation. Although this specimen provides a rare opportunity to characterize advanced HCC after intensive multi-modal therapy (HAIC + TKI + ICI), a single case cannot capture the profound inter-patient heterogeneity of HCC. Therefore, these single-cell findings should be interpreted strictly as exploratory and hypothesis-generating. Second, the proposed links among lactylation-associated transcriptional programs, G6PD-SPP1 coupling, immune suppression, and computationally inferred drug vulnerabilities remain fundamentally correlative. Future functional, lactylation-focused, spatial, and immune-interaction assays are required to establish causality. Finally, because our prognostic signature and protein-level validation were based on retrospective or treatment-naïve surgical cohorts, there remains a conceptual gap between the post-treatment single-cell discovery and treatment-naïve clinical validation. Thus, our study primarily identifies an intrinsically aggressive, high-risk biological state rather than proving a clinically acquired resistance mechanism. The predictive value of the G6PD-SPP1 association for contemporary combination therapies should be evaluated in prospective longitudinal cohorts receiving HAIC, PD-1/PD-L1 blockade, TKIs, or their combinations, using objective response, progression-free survival, overall survival, and acquired resistance as endpoints.

In summary, we describe a metabolically and immunologically distinct aggressive tumor state in HCC characterized by G6PD-mediated PPP activation and context-dependent SPP1-driven remodeling. The five-gene lactylation-related signature stratifies patient outcomes across independent cohorts and is associated with a checkpoint-rich but ineffective immune phenotype. These findings support further investigation of the G6PD-SPP1 association, and its potential value as a candidate therapeutic target, and provide a rationale for biomarker-stratified trials that combine metabolic inhibition with immunotherapy in HCC.

## Data Availability

The datasets presented in this study can be found in online repositories. The names of the repository/repositories and accession number(s) can be found below: https://ngdc.cncb.ac.cn/, PRJCA061545.

## Supplementary material

SUPPLEMENTARY TABLE S1 Differentially expressed genes in hepatocytes. Complete list of DEGs identified in hepatocytes comparing the therapy-progressive lesion with treatment-naïve HCC samples.

SUPPLEMENTARY TABLE S2 Lactylation-related gene set. Curated list of 462 lactylation-related genes from GeneCards and literature sources.

SUPPLEMENTARY TABLE S3 Overlapping candidate genes. List of 53 genes identified by cross-platform integration.

SUPPLEMENTARY TABLE S4 Univariable Cox regression results. Statistical results for 14 genes significantly associated with overall survival.

SUPPLEMENTARY TABLE S5 CellChat-derived cell-group communication probabilities from the specimen-level comparison between the therapy-progressive lesion and treatment-naïve HCC samples.

SUPPLEMENTARY TABLE S6 Clinicopathological characteristics of the surgical cohortBaseline clinical and pathological features of the 114-patient surgical cohort stratified by exploratory X-tile-defined G6PD expression groups.

SUPPLEMENTARY FIGURE S1 Validation of cell-type annotations. Expression of canonical marker genes across annotated cell populations.

SUPPLEMENTARY FIGURE S2 Extended immune characterization and drug response analysis. **(A)** Immune checkpoint gene expression across risk groups. **(B)** Correlation between risk signature genes and predicted drug sensitivity.

SUPPLEMENTARY FIGURE S3 Single-cell characterization of signature activity and communication. **(A)** AUCell score distribution and threshold determination. **(B)** Distribution of signature-high cells across cell types. **(C)** Global intercellular communication strength comparison between signature-high and signature-low cells. **(D)** Pathway-level communication differences between signature-high and signature-low cells, highlighting SPP1. **(E)** SPP1 ligand–receptor interactions between therapy-progressive lesions and treatment-naïve HCC.
